# On‐line preparatory information for children and their families undergoing dental extractions under general anesthesia: A phase III randomized controlled trial

**DOI:** 10.1111/pan.13307

**Published:** 2017-12-27

**Authors:** Corinne Huntington, Christina Liossi, Ana Nora Donaldson, Jonathan Timothy Newton, Patricia A. Reynolds, Reham Alharatani, Marie Therese Hosey

**Affiliations:** ^1^ Division of Population and Patient Health King's College London Dental Institute London UK; ^2^ University of Southampton and Great Ormond Street Hospital for Children NHS Trust Southampton UK; ^3^ Department of Applied Mathematics & Statistics Stony Brook University Stony Brook NY USA

**Keywords:** anesthesia, child, general [*psychology], preoperative care, tooth extraction, video games [psychology]

## Abstract

**Background:**

Family‐centered interactive on‐line games are increasingly popular in healthcare, but their effectiveness for preoperative preparation needs further research. www.scottga.org is the new on‐line version of a proven nonweb‐based game for children and parents/caregivers.

**Aims:**

The aim of this study was to evaluate if www.scottga.org improved children's anxiety and families' satisfaction compared with controls.

**Methods:**

In this phase III double‐blind randomized controlled trial, children/parents/caregivers received (i) www.scottga.org, (ii) standard care, or (iii) a placebo hand‐washing game. The intervention and placebo games were available online for home usage and provided again on the ward before surgery. All children were accompanied by parent/caregivers at induction and observed and scored using validated measures. Stratified randomization and generalized linear models were used. An intention‐to‐treat approach was adopted.

**Results:**

Overall, 52/176 children had baseline “psychological disturbance.” Children's anxiety increased preinduction, but there were no differences between groups (Facial Image Scale: video‐standard OR = 1.08, *P* = .82, 95% CI [0.56, 2.1]; video‐placebo OR = 0.9, *P* = .77 95% CI [0.46, 1.8]). There were no differences in induction behavior (visual analog scale: video mean = 3.5; standard care mean = 3.5; placebo mean = 3.7: video‐standard OR = 2.0, *P* = .42, 95% CI [−0.6, 1.3]; video‐placebo OR = 1.53, *P* = .65, 95% CI [−0.8, 1.1]) or induction anxiety (modified Yale Preoperative Anxiety Scale: video‐standard OR 1.02, *P* = .97, 95% CI [0.61, 2.6]; video‐placebo OR 1.38, *P* = .49, 95% CI [0.87, 3.81]). Families favored the intervention regarding the “child handling the visit better” (Treatment Evaluation Inventory: video‐standard OR = 12; 95% CI 4.7‐32; *P* < .001; video‐placebo OR = 8.2; 95% CI 3‐22; *P* < .001) and “improving the child's ability to cope” (Treatment Evaluation Inventory: video‐standard OR = 21; 95% CI 8‐56; *P* < .001 and video‐placebo OR = 13; 95% CI 5‐34; *P* < .001).

**Conclusion:**

Families believed that a video‐game preparation helped their child's perioperative anxiety, but there were no objective measures of behavioral improvement associated with this intervention.

**What is already known**

- On‐line “serious” games are increasingly used for patient education, information, and psychological. Children need time to rehearse their coping strategies at home in advance. A previously published computer game, given to children and their parent/carer before being taken to induction, proved to help the children to cope. Therefore, to help parents/carers to prepare their children at home, http://www.scottga.org is a new on‐line game that builds upon that intervention.

**What this article adds**

- Using http://www.scottga.org neither caused nor improved anesthetic induction anxiety and families believed that the game helped their children to cope better with the GA experience.

## 1 INTRODUCTION

Tooth decay is the commonest disease of childhood worldwide, in the United Kingdom (UK), tooth extraction under general anesthesia (GA) is the commonest reason for child hospital admission, with 60 683 children admitted in England in 2012/13.^1^ The GA is part of a short‐case hospital day surgery procedure lasting approximately 10 minutes. The child is rendered completely unconscious, usually by gas induction, and nasal or laryngeal masks are used for maintenance. These are healthy children (ASAI); those with medical/learning disabilities are admitted under a different service. A typical operatory list includes only this cohort and numbers 8‐10 children. The children are commonly aged around 6 years and scheduled to have an average of 7 primary teeth removed. Approximately 20% of their parents are dentally anxious.^2^ The Association of Paediatric Anaesthetists of Great Britain and Ireland (2011) have recommended offering these children preoperative nonpharmacological preparation.^3^ Computer games, role‐modeling, parental coaching, coping skill instruction, clowns/clown‐doctors, and procedural information have all been reported in the literature, but a Cochrane Review has shown that these studies have a high risk of bias and has recommended more research.^4^, ^5^, ^6^, ^7^, ^8^, ^9^, ^10^, ^11^, ^12^ A videogame, given to children and parents/carers immediately prior to entering the induction room, was found to alleviate perioperative anxiety; it was tested on a sample of 198 children scheduled for tooth extraction who were allocated to game, placebo‐cartoon or to blank control groups in a phase II RCT^11^ and included in the aforementioned Cochrane review.^4^

Children need time to rehearse their coping strategies^6^, ^12^ and so parents/carers need to be able to prepare their children at home. Therefore, on‐line serious games^9^, ^13^ might address this need. Moreover, the UK's National Health Service recognizes the therapeutic benefit of on‐line psychological interventions.^14^ Therefore, a new on‐line game that built upon the videogame that was tested in the aforementioned RCT^11^ was developed through focus groups, literature review, expert consultation and field‐testing and is freely available at http://www.scottga.org.

The aim of this study is to compare www.scottga.org to 2 controls: (i) standard care and (ii) a placebo video game, in terms of child behavior and anxiety; family satisfaction and reduction in induction and discharge times. The hypotheses are that the game will reduce children's preoperative anxiety, improve family satisfaction, and reduce induction and discharge times.

## 2 MATERIALS AND METHODS

In line with guidance recommending the publication of study protocols prior to the completion of data collection and analysis, the study protocol was published in a peer‐reviewed journal.^15^ This is a prospective, 3‐armed double‐blind, randomized, phase III trial, carried out in the Day Surgery Unit (DSU) of King's College Hospital, London, England. It was undertaken in accordance with the Declaration of Helsinki. All parents and children gave their written consent (NHS REC: 10/H0802/41: R&D‐ KCH 11‐024). Validated measures were used throughout. The participating family and child, the clinical researcher; the statistician, the anesthetist and dental surgeon; and the DSU team, including the nurses inputting the routine NHS throughput time data, were blind to the allocation. The blinded clinical researcher collected all of the primary outcome measures on the day of the GA: observed child anxiety/distress at induction; child perioperative self‐reported anxiety; and family satisfaction with the preparatory material and with the service. An unblinded researcher, recruited the families and children, delivered information packs, and conducted the postoperative telephone‐structured interviews, which included (i) postoperative child anxiety and (ii) family satisfaction with both (a) the preparatory material and (b) the service.

**Figure 1:**
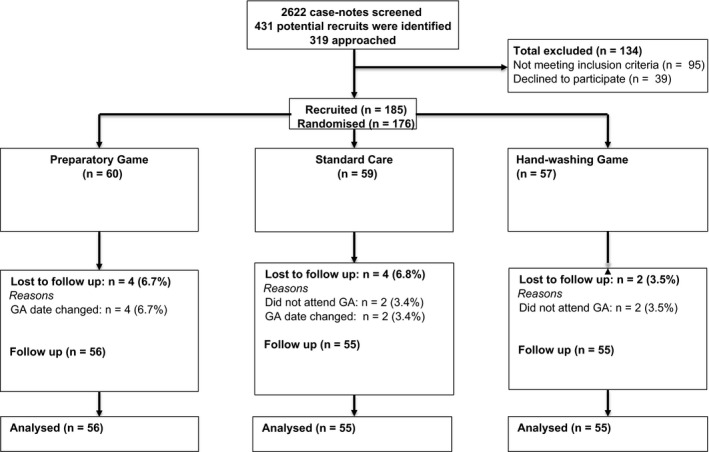
Consort flow chart

Families of 5‐ to 7‐year‐old children scheduled for GA tooth extraction were invited to participate. Since this type of GA procedure is routinely limited to ASAI children, those with known medical or behavioral conditions were already excluded. Also excluded were families reporting insufficient Internet capability for “You‐Tube,” and children who had already experienced a GA when older than 2 years of age. Recruitment occurred on the anesthetic assessment clinic between July 2012 and December 2013, about 2 weeks in advance of the GA visit. Of 185 consenting families, 176 were randomized (Figure 1). Nine families were excluded due to their child's previously undisclosed learning disability or previous GA; they were all offered the on‐line video game without data collection.

### 2.1 Randomization

Randomization by minimization on gender and age group (5‐7 years of age) with a 1:1:1 allocation ratio was performed by the blinded trial statistician. The unblinded researcher delivered identical packs in a predetermined order. Opening them revealed the allocation. Curtains around the beds prior to surgery maintained allocation concealment during the DSU admission.

### 2.2 Interventions

1. The intervention‐video group were provided with their own unique on‐line access plus standard care (fasting and wound care instruction). www.scottga.org has 22 screens that families interact with by “mouse‐clicking” and contains a cartoon “story,” 2 videos which model appropriate behavior and teach coping skills. An accompanying pamphlet directed parents/carers about how to use it with their child. They were prompted via e‐mail to access it approximately 1 week before the operation and child/parents were given it again on the ward on arrival for the surgery. Each family's unique URL identifier enabled their usage to be recorded.
2. The standard‐care group received a pack with fasting and wound care instruction plus a coloring book (about healthy food choices).
3. The placebo‐video group received the same standard care materials plus access information to a hand‐washing video of similar length and target age to the intervention. They were reminded via e‐mail to access it approximately 1 week before the operation and were given it again on the ward on arrival for the surgery.

### 2.3 Descriptive data and measures

- Family profile and parental education, child dental anxiety (a score of 18 and above on the MCDAS),^16^ psychological status^17^ details of the procedure (number and type of teeth removed, anesthetic agent and local and systemic analgesia) were recorded.
- Each child self‐reported their anxiety using the Facial Image Scale (FIS)^18^ at recruitment, on arrival on the DSU ward and again 48 hours and 1 week postoperatively.
- Observed child anxiety was recorded immediately before entry and again inside the anesthetic induction room using the modified Yale Preoperative Anxiety Scale (m‐YPAS).^19^ Observed child behavior during the anesthetic induction was scored using a 10‐cm visual analog scale^20^ (VAS, 10 equaled worst behavior). The induction was video recorded to (i) improve data capture, so that video footage could be used whenever direct observation could not occur and (ii) evaluate intra‐rater and inter‐observer reproducibility. The blinded researcher, re‐scored 36 videos using the VAS, yielding an intra‐observer reproducibility (intra‐class correlation: ICC) of 0.87 (95% CI 0.79‐0.95). A second blinded clinical observer scored the same videos (inter‐observer) ICC = 0.77 (95% CI 0.63‐0.92). Bland‐Altman plots confirmed the intra‐ and inter‐observer reproducibility.
- Parents rated their satisfaction with both the hospital service and the preparatory information received on 2 occasions. First, immediately prior to discharge from the ward using a VAS^21^ (satisfaction equaled a score of 5‐10) in person to the blinded researcher who was not introduced to them as member of the research team. Second, 48 hours later, by telephone, using the Treatment Evaluation Inventory (TEI)^22^ to the un‐blinded researcher.
- Service throughput was timed in minutes as follows: (i) induction time, from entering the anesthetic induction room to transfer into the operating room; (ii) operating time, from entry into the operating room to exit to the recovery room; (iii) time in the recovery room, from entry to the recovery room to exit to the ward; (iv) time in the ward, from entry from the recovery room to discharge home. These data are automatically recorded as part of routine NHS monitoring Operating room management system (Galaxy (c) CFC) and are inputted by the regular operating room nurses, who were blind to the allocation; its correctness and validity was verified by the researchers.

### 2.4 Sample size

The sample size calculation was based on the baseline summaries observed in the phase II study^11^ for outcome “child's observed behavior at GA induction.” The study was designed to have 80% power, at the 5% significance level, to detect a difference of 2 on the 10‐cm VAS between the intervention‐video and each of the 2 controls. Anticipating a 3.6 standard deviation, this corresponded to a 0.55 effect size. For this purpose, a sample of 53 children with complete data in each group was sought. Anticipating a 20% drop out rate, the study sought to recruit 67 subjects per group. The effect size 0.55 falls onto the medium‐size range of the Cohen's scale, guaranteeing the intended power for the other (normally distributed) primary outcomes. (This covers the binary outcomes given the one‐to‐one correspondence with their approximately normally distributed log‐odds).

### 2.5 Statistical analysis

An intention‐to‐treat approach was followed. Baseline summaries by group were used to check that they were well balanced by all potential prognostic variables. Comparisons between the groups in terms of the primary outcomes were performed using linear, ordinal, and logistic regressions for normal, ordinal, and binary outcomes, respectively. The corresponding multivariate models were used to double check for predetermined possible confounders (waiting time preoperatively and number of teeth extracted). Nonparametric models are used when required. To evaluate the longitudinal change in outcomes with repeated‐measures baseline levels were adjusted for. Complete case analysis was adopted with an evaluation of the effect of missing data in the results.

## 3 RESULTS

Of 206 children identified in advance for randomization, 30 did not present for the operation or were ineligible. Of the 176 eligible children, there was complete data in the outcomes measured in the ward for 55 children in the “game” group (of 69 randomized), 56 in the standard care group (of 69 randomized) and 55 in the placebo video game group (of 68 randomized). Therefore, the group numbers ensured the number of children with complete data in the DSU ward‐based outcomes met the intended power requirement. The 10 “drop‐outs” were children who did not undergo the GA procedure whatsoever or were treated out‐with the study period (eg, their operation was rescheduled to a later date); these were as follows: 4 (6.7%) in the intervention, 4 (6.7%) in standard care, and 2 (3.5%) in the placebo‐video game. Drop‐out was not dependent on age, teeth to be extracted or baseline psychological (Rutter)^17^ or dental anxiety (MCDAS)^16^ scores (these were the only variables examined for this group since all the other demographic data were to be collected during the telephone follow‐up).

The randomization balanced the groups by sample size, demographic characteristics, family background and education, and dental and anesthetic procedures as well as for other clinical characteristics (eg, whether the child was dentally anxious). The intervention‐video group appeared to have slightly more children whose scores indicated “psychological disturbance of clinical significance” (scores above 11) but this was not statistically significant (Kruskal‐Wallis test, *P* = .14); nevertheless the effect of this variable was explored in all models. Regarding intervention usage, 23 families accessed it at home and 12 of these used it again on the ward; a further 31 used it on the ward only. Only 3 did not use it at all.

One hundred seventeen children were directly observed and scored at induction; for the remainder, the video footage was used to score. All of the children were accompanied into the anesthetic induction (mother, n = 123; father, n = 45; another adult, n = 7). All had been accompanied by at least one parent/carer on the ward when given their intervention again there. Most were induced (n = 168) and then maintained (n = 172) with a combination of sevoflurane and/or nitrous oxide and oxygen and commonly a laryngeal mask was inserted. An average of 6 teeth (per‐child) were extracted, for 168 children, these were primary teeth. Local analgesia was administered (2% Xylocaine with 1:80 000 adrenaline), mainly into the papilla between the teeth to avoid excessive numbness on waking. Analgesics were given to 102 (58.6%) children, and this was usually intravenous Paracetamol given during the surgery, with Ibuprofen available on request later on the ward. Fentanil/Alfentanil was given to 42 (18%) children. Full details of baseline and surgical operatory procedures can be found in Table 1.

**Table 1:** Participant baseline characteristics

	Intervention video game N = 60	Standard care N = 59	Placebo video game N = 55
Child age			
Mean (SD); number in each age band	6 (0.80); 20; 19; 21	6 (0.83); 18; 21;20	6 (0.80); 18; 20; 17
Gender, boys N (%)	29 (48%)	29 (49%)	28 (51%)
Parent report of child anxiety (MCDAS)			
Mean (SD); range	2.8 (0.82); 1.1‐5	3.0 (0.78); 1‐4.1	2.9 (0.85); 1‐4.6
“Normal”	N = 16 (27.6%)	N = 12 (20.3%)	N = 18 (32.7%)
“Anxious”	N = 38 (65.5%)	N = 40 (67.8%)	N = 29 (52.7%)
“Highly fearful”	N = 4 (6.9%)	N = 7 (11.9%)	N = 7 (12.7%)
Psychological status (Rutter) at baseline			
Mean (SD); range	9.5 (5.8); 0‐28	7.46 (4.7); 0‐26	8.9 (5.8); 0‐23
Clinically significant psychological disturbance	N = 22 (36.7%)	N = 12 (20.3%)	N = 20 (36.4%)
Parent age‐group			
19‐24	3 (5%)	1 (1.7%)	1 (1.8%)
25‐29	8 (13.3%)	10 (16.9%)	6 (10.9%)
30‐34	15 (25%)	9 (15.3%)	10 (18.2%)
35‐39	12 (20%)	14 (23.7%)	12 (21.8%)
40+	19 (31.7%)	14 (23.7%)	12 (21.8%)
Parent qualification			
None	7 (11.7%)	4 (6.8%)	2 (3.5%)
GCSE/O‐Level	13 (21.7%)	4 (6.8%)	10 (17.5%)
A levels	1 (1.7%)	5 (8.5%)	8 (14%)
Diploma/NVQ	16 (26.7%)	20 (33.9%)	11 (19.3%)
University degree	14 (23.3%)	11 (18.6%)	6 (10.5%)
Postgraduate degree	6 (10%)	4 (6.8%)	3 (5.3%)
Parenting status			
Single	10 (16.7%)	15 (25.4%)	8 (14.5%)
Married	30 (50%)	22 (37.3%)	22 (40%)
Living together	9 (15%)	5 (8.5%)	6 (10.9%)
Living apart	7 (11.7%)	3 (5.1%)	5 (9.1%)
Parent with other	1 (1.7%)	4 (6.8%)	0 (0%)
Number of teeth extracted. Mean (SD); range	6.45 (2.5); 1‐14	6.05 (2.5); 1‐12	6.92 (2.2); 2‐14
Preoperative toothache (Bieri)			
Mean (SD)	1.9 (2.9); 0‐10	2.3 (3.5); 0‐10	1.56 (2.5); 0‐10
“In Pain”	9 (14.9%)	14 (23.8%)	7 (12.7%)
GA induction (gaseous) N (%)	59 (98.3%)	56 (94.9%)	53 (96.4%)
Sevoflurane usage N (%)	60 (100%)	58 (98.3%)	55 (100%)
Dose of local anesthetics (mL) Mean (SD); range	1.62 (0.65); 0.55‐3.3	1.35 (0.7); 0.55‐3.3	1.8 (0.7); 0.55‐4.4
Children given perioperative analgesics N (%)	59 (98.3%)	59 (100%)	51 (92.7%)
Waiting time (minutes) on the ward			
Mean; range (95% CI)	95.8; 15‐90 (85, 107)	77.2; 21‐197 (67, 88)	89.7; 25‐174 (80, 100)
Time (minutes) in recovery room			
Mean; range (95% CI)	17.2; 6‐61 (14.5, 19.8)	16.7; 6‐37 (15, 18.5)	15.3; 6‐34 (13.8, 17)

No significant difference was found between the intervention‐video and the 2 controls regarding the child's behavior at induction. Regarding their anxiety before going into, and within, the induction room immediately before anesthetic induction, recorded by the m‐YPAS; on linear regression (for the normal outcome) and, on logistic regression for the likelihood of a high YPAS (above 31 for the former and above 25 for the latter), no significant difference was found between the intervention‐video and the 2 controls. Using Kruskal‐Wallis, no significant difference between the groups was found regarding anesthetic induction duration, recovery time or time to discharge. Further results, including odds ratios, are detailed in Table 2. On logistic regression, the likelihood of requesting an analgesic postoperatively was also independent of the group (*P* = .89).

**Table 2:** Anesthetic induction behavior, anxiety and service throughput

Outcome	Intervention video N = 55	Standard care N = 56	Placebo video N = 55
Behavior at anesthetic induction (VAS)			
Mean (SD); range	3.5 (2.6); 0‐10	3.5 (2.5); 0‐9.5	3.7 (2.4); 0‐10
Linear regression (coefficient relative to controls)			
Coefficient; 95% CI; *P*‐value		0.35; (−0.6, 1.3); *P* = .46	0.15; (−0.8, 1.1); *P* = .75
Logistic regression (likelihood of VAS = 4+)			
Intervention video relative to controls OR (95% CI)		2.0; (0.36‐11.6)	1.53; (0.25, 9.5)
*P*‐value		*P* = .42	*P* = .65
Child distress/anxiety (YPAS) at presentation for induction			
Mean (SD); range	47.6 (22.2); (23‐100)	45.1 (20.5); (22.9‐100)	43.2 (20.7); (22.9‐95.8)
Intervention video relative to controls OR (95% CI)		1.26 (0.61‐2.6)	1.82; (0.87‐3.81)
*P*‐value		*P* = .53)	*P* = .11
Child distress/anxiety (YPAS) at induction			
Mean (SD); range	47.6 (22.2); (23‐100)	45.1 (20.5); (22.9‐100)	43.2 (20.7); (22.9‐95.8)
Intervention video relative to controls OR (95% CI)		1.02; (0.40‐2.6)	1.38; (0.56, 3.4)
*P*‐value		*P* = .97	*P* = .49
Anesthetic induction duration (minutes)			
Mean (SD); range	9.33 (5.5); 2‐34	9.2 (5.1); 3‐7	9.5 (4.3); 0‐21
Time (minutes) on ward after recovery			
Mean (SD); range	47 (25); 15‐123	55 (31); 14‐217	49 (22); 20‐120

OR, odds ratio.

The child's self‐reported anxiety^18^ was recorded as an ordinal variable with 5 levels (from 1: “relaxed/not worried” to 5 indicating “extremely worried”). Table 3 shows the trend of this outcome over the 4 time points (recruitment, on presentation on the ward preoperatively, and 2 postoperative) by group, including the point estimate and 95% CI for the odds ratio of worse anxiety level for the intervention‐video game in relation to the 2 control groups. Anxiety was at its worst, while the children were waiting on the ward just before being taken to the induction room. On ordinal logistic regression, no significant difference was found between the intervention and controls, at any of the time points.

**Table 3:** Counts and ordinal logistic regression results for child self‐reported anxiety (FIS)

Variable	Treatment group			Sig^a^
	Video game (N = 60)	Standard care (N = 59)	Placebo video game (N = 55)	
Anxiety‐recruitment‐FIS				
1. Relaxed/no‐worried	38 (63.0%)	38 (64.4%)	36 (65.5%)	
2. Worried little	9 (15.0%)	13 (22.0%)	12 (21.8%)	
3. Fairly worried	11 (18.3%)	7 (11.9%)	7 (12.7%)	
4. Worried lot	0	1 (1.7%)	0	
5. Extremely worried	2 (3.3%)	0	0	
Video game relative to controls OR (95% CI); *P*‐value		1.18 (0.57, 2.5) *P* = .65	1.25 (0.59, 2.7) *P* = .56	0.82
Anxiety‐“on the ward” preoperative‐FIS				
1. Relaxed/no‐worried	24 (40.0%)	25 (42.4%)	24 (43.6%)	
2. Worried little	10 (16.7%)	12 (20.3%)	4 (7.3%)	
3. Fairly worried	15 (25.0%)	9 (15.3%)	15 (27.3%)	
4. Worried lot	4 (6.7%)	6 (10.2%)	3 (5.5%)	
5. Extremely worried	7 (11.7%)	7 (11.9%)	9 (16.4%)	
Video game relative to controls OR (95% CI); *P*‐value		1.08 (0.56, 2.1) *P* = .82	0.90 (0.46, 1.8) *P* = .77	0.91
Anxiety‐48‐h postoperative‐FIS				
1. Relaxed/no‐worried	32 (62.8%)	31 (67.4%)	33 (73.3%)	
2. Worried little	5 (9.8%)	9 (19.6%)	6 (13.3%)	
3. Fairly worried	7 (13.7%)	2 (4.4%)	5 (11.1%)	
4. Worried lot	3 (5.9%)	2 (4.4%)	0	
5. Extremely worried	4 (7.8%)	2 (4.4%)	1 (2.2%)	
Video game relative to controls OR (95% CI); *P*‐value		1.31 (0.57, 3.0) *P* = .52	1.73 (0.73, 4.1) *P* = .22	0.35
Anxiety‐1‐wk postoperative‐FIS				
1. Relaxed/no‐worried	48 (82.8%)	38 (77.6%)	36 (87.8%)	
2. Worried little	5 (8.6%)	8 (16.3%)	2 (4.9%)	
3. Fairly worried	4 (6.9%)	1 (2.0%)	2 (4.9%)	
4. Worried lot	1 (1.7%)	1 (2.0%)	1 (2.4%)	
5. Extremely worried	0	1 (2.0%)	0	
Video game relative to controls OR (95% CI); *P*‐value		0.54 (0.20, 1.49) *P* = .23	1.31 (0.39, 4.4) *P* = .66	0.51

OR, odds ratio.

a Result of the ordinal logistical regression model.

Every family reported high levels of satisfaction at discharge to the blinded researcher. Table 4 shows the summaries, results of the logistic regression modeling and the odds ratios of the likelihood of having a top score (9 or 10 on the VAS) for satisfaction reported to the blinded observer.^21^ No significant differences between the intervention and the controls were found for either “satisfaction with the hospital service” (*P* = .95), “satisfaction with the preparatory information” (*P* = .71) or “usefulness of the preparatory information” (*P* = .63). At the 48‐hour telephone call, with the nonblind researcher applying the 6 items of the *Treatment Evaluation Inventory* (TEI),^22^ on logistic regression, no significant difference between the groups was found regarding “how the child will handle future GA” and “how the preparatory information helped the child” but highly significant differences were detected between the intervention and the controls, in terms of the 2 items scoring parents' perception of how the preparation helped the child's GA experience. The odds ratio for likelihood of “a lot/very much satisfaction” of the intervention relative to standard care and placebo‐video was in favor of the intervention. They were, respectively, OR = 12; 95% CI 4.7‐32; *P* < .001 and OR = 8.2; 95% CI 3‐22; *P* < .001, for TEI‐1 “Do you think the information you received helped your child to handle the visit better?”, and OR = 21; 95% CI 8‐56; *P* < .001 and OR = 13; 95% CI 5‐34; *P* < .001, for TEI‐2 “Do you think the information improved your child's ability to cope?”

**Table 4:** Satisfaction at the time of discharge (VAS 0‐10). Summaries and logistic regression results for likelihood of essentially complete satisfaction (VAS = 9 or 10)

Satisfaction with	Intervention video game	Standard care	Placebo video game
Hospital service			
Mean (SD); range	8.2 (2.5); 0‐10	8.8 (1.2); 6‐10	8.8 (1.5); 4‐10
Logistic regression for score 9‐10			
Video game relative to controls OR (95% CI)		1.11 (0.5, 2.5)	1.13 (0.5, 2.6);
*P*‐value		*P* = .79	*P* = .77
Preparatory information			
Mean (SD); range	8.8 (1.9); 0‐10	9.0 (1.0); 5‐10	8.9 (1.6); 4‐10
Logistic regression for score 9‐10			
Video game relative to controls OR (95% CI)		1.5 (0.6, 3.74)	1.4 (0.54, 3.73)
*P*‐value		*P* = .45	*P* = .48
Preparatory information assisting parent to prepare child			
Mean (SD); range	8.8 (2.0); 0‐10	9.0 (1.0); 6‐10	8.9 (1.4); 3.5‐10
Logistic regression for score 9‐10			
Video game relative to controls OR (95% CI)		1.1 (0.46, 2.8)	1.5 (0.62, 3.7)
*P*‐value		*P* = .78	*P* = .36

OR, odds ratio.

### 3.1 Missing data

Missing data were only present in the 48‐hour home follow‐up for the TEI measure. It was not substantial and was irrespective of group: only 13 children (7.6%) had missing data overall: 2 (3%) in the intervention, 5 (8.5%) in standard care and 6 (11%) in the placebo group. It was not dependent on age, gender (no other socio‐demographic data were collected for these 13 children), or any other baseline clinical characteristic. On this basis, the missing data in the TEI outcomes are classified as missing completely at random (MCAR) for which a complete case analysis is a valid approach.

### 3.2 Patient and public involvement

Before this study, 56 child‐GA‐tooth‐extraction families gave us feedback on the study design; over 70% of them reported ownership of a computer with Internet access sufficient to support “You‐Tube” and on‐line games. They suggested how this “serious” game might be utilized. Eleven more families fed‐back on the content of www.scottga.org and also about using video‐recording during anesthetic induction.

## 4 DISCUSSION

This study evaluated the utility of an on‐line family‐centered preparation for children scheduled for GA for tooth extraction, namely www.scottga.org in decreasing perioperative anxiety and improving satisfaction. The findings suggest that the online game did not cause preoperative anxiety and that families liked being offered preparation in this form and believed that it helped their children to cope better with the hospital event. However, it neither reduced the children's perioperative anxiety nor lead to faster induction or discharge. None of the preparatory materials served to distract or allay the child's anxiety immediately before being taken into the induction room.

This study raises questions about the efficacy of www.scottga.org and of “stand‐alone” on‐line preoperative “self‐help” style interventions generally; especially when they are delivered without other support for the parents/carers at home and without interventions for the child immediately before and inside the induction room. Scottga.org mainly provided information and modeling of coping, but coping styles vary, and the content may not have been tailored enough to meet the needs of those few children who were noncooperative. Similar studies into home‐based family preparation have additionally included behavioral skill coaching^7^ to the parents, while others have tailored their intervention to their child's preferred coping style.^9^ Other researchers have shown that children also benefit by playing their choice of videogame inside the induction room.^10^

Almost all the families who were allocated to the intervention group used www.scottga.org, either at home or on the ward, but less than half chose to access it at home despite prompting. This is unlikely to be due to difficulties with Internet access, since the UK has comparatively high access, even among lower socio‐economic groups, and especially in cities such as London. This meant that many of the children did not have enough time to rehearse, so could not benefit fully. Other researchers have recently tested an on‐line intervention (WebTIPS) that included tailored parent/carer coaching by telephone; and in a preliminary RCT of 82 (38 intervention group) children aged 2‐7 years undergoing mainly tonsillectomy and adenoidectomy, they reported that the intervention children were less anxious than controls prior to and during induction, although all of the children's anxiety increased when the anesthetic mask was introduced.^9^ Thus, in future, it might be best to explore combinations of: on‐line preparation, family coaching at home, and the child's choice of videogame just immediately before and inside the induction room. However, in a short‐case list, such as the one that was studied presently, this combination might prove costly. Therefore, future research should also include a cost‐benefit analysis.

We focused on children with severe tooth decay not only because this is the main reason for UK pediatric GA admissions but also because it achieved a homogenous sample and standardized the surgery and the day surgery processes. Other studies have sampled broader age ranges, fewer subjects, wealthier families and mixtures of procedures. In this study, two‐thirds of the children were “anxious” and a third had emotional and behavioral difficulties. Increased levels of dental anxiety and poor psychological well‐being have been reported before in this type of patient.^23^ Recruiting hard‐to‐reach families into pediatric clinical trials is challenging, but failing to do so can result is biased reporting since studies might not reflect the population as a whole. Therefore, it is a strength of the present study to have recruited and retained these challenging children and their families. However, tooth‐decay is linked to poor socio‐economic status which in turn is known to impact on families' engagement with services and support.^23^, ^24^, ^25^ As such, our study may not be generalizable to wealthier or to better‐educated families.

The families' satisfaction with all of the preparations, reported to the blinded researcher on discharge, was high but is similar to other studies that used this measure.^21^ The scripted telephone interviews suggest that the families believed that www.scottga.org helped their child to cope better but this finding needs to be cautiously interpreted given the unblinded nature of the data acquisition and the subjective nature of the outcome measure.^15^, ^22^, ^25^

## 5 CONCLUSION

The on‐line preparation www.scottga.org did not improve the children's perioperative anxiety or behavior or lead to shorter induction and discharge times compared with controls. In spite of this finding, families believed that the application helped their child to cope with and handle the GA experience better. More work is required to determine the ideal method for using online preparation tools for preoperative preparation.

## ETHICAL APPROVAL

South East London Research Ethics Committee 2 (10/H0802/41).

## CONFLICT OF INTEREST

The authors report no conflict of interest.
